# (*E*)-1-(4-Amino­phen­yl)-3-(naphthalen-2-yl)prop-2-en-1-one

**DOI:** 10.1107/S1600536811014024

**Published:** 2011-04-22

**Authors:** Thawanrat Kobkeatthawin, Suchada Chantrapromma, Nisakorn Saewan, Hoong-Kun Fun

**Affiliations:** a Crystal Materials Research Unit, Department of Chemistry, Faculty of Science, Prince of Songkla University, Hat-Yai, Songkhla 90112, Thailand; b School of Cosmetic Science, Mae Fah Luang University, Muang, Chiang Rai 57100, Thailand; c X-ray Crystallography Unit, School of Physics, Universiti Sains Malaysia, 11800 USM, Penang, Malaysia

## Abstract

The mol­ecule of the title chalcone derivative, C_19_H_15_NO, exists in a *trans* configuration with respect to the C=C double bond. The mol­ecule is slightly twisted with a dihedral angle of 6.12 (12)° between the benzene ring and the naphthalene ring system. The prop-2-en-1-one bridge is nearly planar, with an r.m.s. deviation of 0.0194 (2), and makes dihedral angles of 8.05 (19) and 11.47 (18)° with the benzene ring and the naphthalene ring system, respectively. In the crystal, mol­ecules are linked by N—H⋯O hydrogen bonds into chains along the *b* axis. Weak N—H⋯π and C—H⋯π inter­actions and a short N⋯O contact [2.974 (4) Å] are also observed.

## Related literature

For bond-length data, see: Allen *et al.* (1987 ▶). For related structures, see: Fun *et al.* (2008 ▶); Horkaew *et al.* (2010 ▶). For background to and applications of chalcones, see: Bandgar & Gawande (2010 ▶); Cheng *et al.* (2008) ▶; Gaber *et al.* (2008 ▶); Nerya *et al.* (2004 ▶); Nowakowska *et al.* (2008 ▶); Patil *et al.* (2007 ▶); Svetlichny *et al.* (2007 ▶); Tewtrakul *et al.* (2003 ▶); Xu *et al.* (2005 ▶). For the stability of the temperature controller used in the data collection, see Cosier & Glazer, (1986 ▶).
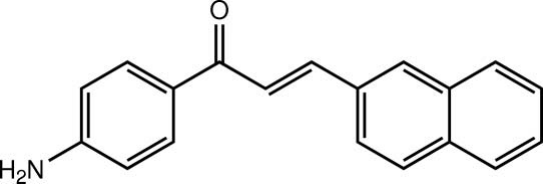


## Experimental

### 

#### Crystal data

C_19_H_15_NO
*M* *_r_* = 273.32
Orthorhombic, 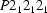

*a* = 5.7422 (6) Å
*b* = 9.8022 (10) Å
*c* = 25.504 (3) Å
*V* = 1435.5 (3) Å^3^
*Z* = 4
Mo *K*α radiation
μ = 0.08 mm^−1^
*T* = 100 K
0.32 × 0.28 × 0.07 mm

#### Data collection

Bruker APEX DUO CCD area-detector diffractometer
Absorption correction: multi-scan (*SADABS*; Bruker, 2009 ▶) *T*_min_ = 0.976, *T*_max_ = 0.994
8109 measured reflections
1940 independent reflections
1633 reflections with *I* > 2σ(*I*)
*R*_int_ = 0.042

#### Refinement

*R*[*F* ^2^ > 2σ(*F* ^2^)] = 0.057
*wR*(*F* ^2^) = 0.119
*S* = 1.14
1940 reflections
198 parameters
H atoms treated by a mixture of independent and constrained refinement
Δρ_max_ = 0.29 e Å^−3^
Δρ_min_ = −0.24 e Å^−3^

### 

Data collection: *APEX2* (Bruker, 2009 ▶); cell refinement: *SAINT* (Bruker, 2009 ▶); data reduction: *SAINT*; program(s) used to solve structure: *SHELXTL* (Sheldrick, 2008 ▶); program(s) used to refine structure: *SHELXTL*; molecular graphics: *SHELXTL*; software used to prepare material for publication: *SHELXTL* and *PLATON* (Spek, 2009 ▶).

## Supplementary Material

Crystal structure: contains datablocks global, I. DOI: 10.1107/S1600536811014024/rz2579sup1.cif

Structure factors: contains datablocks I. DOI: 10.1107/S1600536811014024/rz2579Isup2.hkl

Additional supplementary materials:  crystallographic information; 3D view; checkCIF report

## Figures and Tables

**Table 1:** Hydrogen-bond geometry (Å, °) *Cg*1, *Cg*2 and *Cg*3 are the centroids of the C1–C6, C10–C12/C17–C19 and C12–C17 rings, respectively.

*D*—H⋯*A*	*D*—H	H⋯*A*	*D*⋯*A*	*D*—H⋯*A*
N1—H1*N*1⋯O1^i^	0.90 (4)	2.12 (4)	2.974 (4)	159 (4)
N1—H2*N*1⋯*Cg*1^ii^	0.86 (4)	2.99 (4)	3.475 (3)	118 (3)
C5—H5*A*⋯*Cg*3^iii^	0.93	2.82	3.513 (3)	132
C11—H11*A*⋯*Cg*3^iv^	0.93	2.92	3.631 (3)	135
C13—H13*A*⋯*Cg*2^iv^	0.93	2.86	3.551 (3)	132
C16—H16*A*⋯*Cg*1^iii^	0.93	2.87	3.603 (4)	136

Symmetry codes: (i) 
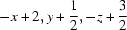
; (ii) 
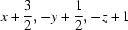
; (iii) 
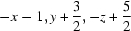
; (iv) 
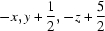
.

**Fig. 1.**
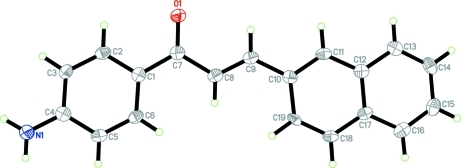
The molecular structure of the title compound, showing 50% probability displacement ellipsoids.

**Fig. 2.**
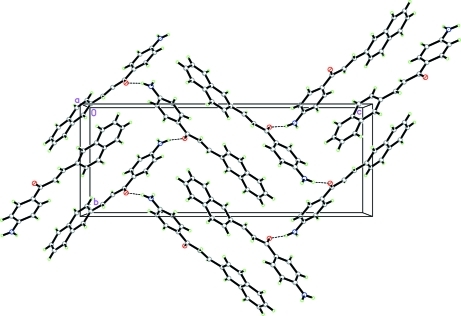
The crystal packing of the title compound viewed along the a axis, showing chains running along the [010] direction.

## supplementary crystallographic information

### Comment

Chalcones are the precursors of flavonoids and antiflavonoids which are available in plenty in ferns and higher plants. Their derivatives are known to display a variety of biological properties such as analgesic, anti-inflammatory, antibacterial and antifungal activities (Bandgar & Gawande, 2010; Cheng *et al.*, 2008; Nowakowska *et al.*, 2008), HIV-1 protease inhibitory (Tewtrakul *et al.*, 2003) and tyrosinase inhibitory (Nerya *et al.*, 2004) activities. Moreover, some of them have also been studied for fluorescent property (Gaber *et al.*, 2008) and used for sensor, liquid crystal display and fluorescence probe for sensing of DNA or proteins (Svetlichny *et al.*, 2007; Xu *et al.*, 2005). In addition, some of them exhibit second harmonic generation (SHG), and hence are used in non-linear optical (NLO) applications (Patil *et al.*, 2007). These interesting properties of chalcones have lead us to synthesize the title compound, (I), which contains the amino and fluorophore (naphthalene) groups in order to study its NLO and fluorescent properties. Compound (I) crystallizes in the chiral orthorhombic *P*2_1_2_1_2_1_ space group and should therefore exhibit second-order nonlinear optical properties. (I) also shows fluorescent emission at 440 nm when excited at 380 nm. Our results also showed that (I) was inactive for tyrosinase inhibitory. Herein the crystal structure of (I) is reported.

The molecule of the title chalcone derivative (Fig. 1), C_19_H_15_NO, exists in an *E* configuration with respect to the C8═C9 ethenyl bond [1.330 (4) Å], as indicated by the torsion angle C7–C8–C9–C10 = 179.1 (3)°. The molecule is slightly twisted with the dihedral angle between the benzene and naphthalene rings of 6.12 (12)°. The prop-2-en-1-one unit (C7—C9/O1) is nearly planar [*r.m.s*. of 0.0194 (2) Å] and the torsion angle O1–C7–C8–C9 is 6.4 (5)°. This middle bridge makes dihedral angles of 8.05 (19) and 11.47 (18) ° with the benzene and naphthalene rings, respectively. The bond distances are of normal values (Allen *et al.*, 1987) and are comparable with those found in related structures (Fun *et al.*, 2008; Horkaew *et al.*, 2010).

In the crystal packing, the molecules are linked by N—H···O hydrogen bonds (Table 1) into chains along the *b* axis (Fig. 2). N—H···π and C—H···π weak interactions (Table 1) are present in the crystal. In addition, a N···O short contact [2.974 (4) Å; symmetry code 2 - *x*, 1/2 + *y*, 3/2 - *z*] is also observed.

### Experimental

The title compound was synthesized by condensation of 4-aminoacetophenone (0.40 g, 3 mmol) with 2-naphthaldehyde (0.46 g, 3 mmol) in ethanol (25 ml) in the presence of 20% NaOH(aq) (5 ml). After stirring for 6 h at room temperature, the resulting yellow solid was collected by filtration, washed with distilled diethylether, dried and purified by repeated recrysallization from acetone. Yellow plate-shaped single crystals of the title compound suitable for X-ray structure determination were recrystalized from acetone by slow evaporation of the solvent at room temperature after several days. M.p. 416–417 K.

### Refinement

Anime H atoms were located in a difference Fourier map and refined isotropically. The remaining H atoms were positioned geometrically and allowed to ride on their parent atoms, with d(C—H) = 0.93 Å and *U*_iso_ = 1.2*U*_eq_(C). The highest residual electron density peak is located at 0.73 Å from C11 and the deepest hole is located at 1.23 Å from C5. A total of 1345 Friedel pairs were merged as there is no significant anomalous dispersion to determine the absolute cofiguration.

### Crystal data

**Table:**

C_19_H_15_NO	*D*_x_ = 1.265 Mg m^−^^3^
*M**_r_* = 273.32	Melting point = 416–417 K
Orthorhombic, *P*2_1_2_1_2_1_	Mo *K*α radiation, λ = 0.71073 Å
Hall symbol: P 2ac 2ab	Cell parameters from 1940 reflections
*a* = 5.7422 (6) Å	θ = 2.2–27.5°
*b* = 9.8022 (10) Å	µ = 0.08 mm^−^^1^
*c* = 25.504 (3) Å	*T* = 100 K
*V* = 1435.5 (3) Å^3^	Plate, yellow
*Z* = 4	0.32 × 0.28 × 0.07 mm
*F*(000) = 576	

### Data collection

**Table:**

Bruker APEX DUO CCD area-detector diffractometer	1940 independent reflections
Radiation source: sealed tube	1633 reflections with *I* > 2σ(*I*)
graphite	*R*_int_ = 0.042
φ and ω scans	θ_max_ = 27.5°, θ_min_ = 2.2°
Absorption correction: multi-scan (*SADABS*; Bruker, 2009)	*h* = −6→7
*T*_min_ = 0.976, *T*_max_ = 0.994	*k* = −9→12
8109 measured reflections	*l* = −32→33

### Refinement

**Table:**

Refinement on *F*^2^	Primary atom site location: structure-invariant direct methods
Least-squares matrix: full	Secondary atom site location: difference Fourier map
*R*[*F*^2^ > 2σ(*F*^2^)] = 0.057	Hydrogen site location: inferred from neighbouring sites
*wR*(*F*^2^) = 0.119	H atoms treated by a mixture of independent and constrained refinement
*S* = 1.14	*w* = 1/[σ^2^(*F*_o_^2^) + (0.0233*P*)^2^ + 1.3561*P*] where *P* = (*F*_o_^2^ + 2*F*_c_^2^)/3
1940 reflections	(Δ/σ)_max_ = 0.001
198 parameters	Δρ_max_ = 0.29 e Å^−^^3^
0 restraints	Δρ_min_ = −0.24 e Å^−^^3^

### Special details

**Table:**

Experimental. The crystal was placed in the cold stream of an Oxford Cryosystems Cobra open-flow nitrogen cryostat (Cosier & Glazer, 1986) operating at 100.0 (1) K.
Geometry. All e.s.d.'s (except the e.s.d. in the dihedral angle between two l.s. planes) are estimated using the full covariance matrix. The cell e.s.d.'s are taken into account individually in the estimation of e.s.d.'s in distances, angles and torsion angles; correlations between e.s.d.'s in cell parameters are only used when they are defined by crystal symmetry. An approximate (isotropic) treatment of cell e.s.d.'s is used for estimating e.s.d.'s involving l.s. planes.
Refinement. Refinement of *F*^2^ against ALL reflections. The weighted *R*-factor *wR* and goodness of fit *S* are based on *F*^2^, conventional *R*-factors *R* are based on *F*, with *F* set to zero for negative *F*^2^. The threshold expression of *F*^2^ > σ(*F*^2^) is used only for calculating *R*-factors(gt) *etc*. and is not relevant to the choice of reflections for refinement. *R*-factors based on *F*^2^ are statistically about twice as large as those based on *F*, and *R*- factors based on ALL data will be even larger.

### Fractional atomic coordinates and isotropic or equivalent isotropic displacement parameters (Å^2^)

**Table:**

	*x*	*y*	*z*	*U*_iso_*/*U*_eq_	
O1	1.1598 (4)	0.7032 (2)	0.85128 (9)	0.0266 (5)	
N1	0.5373 (7)	1.1585 (3)	0.74175 (13)	0.0339 (8)	
H2N1	0.409 (8)	1.195 (4)	0.7515 (15)	0.045 (13)*	
H1N1	0.599 (7)	1.187 (4)	0.7112 (14)	0.033 (11)*	
C1	0.8419 (6)	0.8481 (3)	0.83133 (11)	0.0201 (7)	
C2	0.9516 (6)	0.9000 (3)	0.78648 (12)	0.0224 (7)	
H2A	1.0945	0.8641	0.7763	0.027*	
C3	0.8535 (6)	1.0026 (3)	0.75714 (12)	0.0230 (7)	
H3A	0.9314	1.0356	0.7278	0.028*	
C4	0.6354 (6)	1.0583 (3)	0.77112 (12)	0.0233 (7)	
C5	0.5252 (6)	1.0072 (3)	0.81610 (12)	0.0232 (7)	
H5A	0.3827	1.0434	0.8264	0.028*	
C6	0.6244 (6)	0.9045 (3)	0.84519 (12)	0.0219 (7)	
H6A	0.5466	0.8715	0.8746	0.026*	
C7	0.9597 (6)	0.7405 (3)	0.86224 (13)	0.0222 (7)	
C8	0.8373 (6)	0.6782 (3)	0.90735 (12)	0.0221 (7)	
H8A	0.6818	0.7000	0.9133	0.027*	
C9	0.9441 (6)	0.5920 (3)	0.93962 (11)	0.0198 (7)	
H9A	1.0988	0.5718	0.9321	0.024*	
C10	0.8433 (6)	0.5257 (3)	0.98562 (12)	0.0187 (7)	
C11	0.9577 (6)	0.4164 (3)	1.00866 (11)	0.0204 (7)	
H11A	1.1021	0.3900	0.9956	0.025*	
C12	0.8612 (6)	0.3438 (3)	1.05134 (12)	0.0199 (7)	
C13	0.9733 (6)	0.2294 (3)	1.07446 (12)	0.0234 (7)	
H13A	1.1171	0.2010	1.0617	0.028*	
C14	0.8731 (7)	0.1609 (3)	1.11511 (13)	0.0275 (8)	
H14A	0.9490	0.0864	1.1299	0.033*	
C15	0.6544 (7)	0.2022 (3)	1.13492 (13)	0.0276 (8)	
H15A	0.5864	0.1540	1.1624	0.033*	
C16	0.5423 (7)	0.3123 (3)	1.11405 (12)	0.0263 (7)	
H16A	0.3998	0.3395	1.1279	0.032*	
C17	0.6402 (6)	0.3855 (3)	1.07164 (11)	0.0196 (7)	
C18	0.5282 (6)	0.4987 (3)	1.04803 (12)	0.0220 (7)	
H18A	0.3853	0.5277	1.0611	0.026*	
C19	0.6240 (6)	0.5658 (3)	1.00685 (12)	0.0211 (7)	
H19A	0.5453	0.6395	0.9922	0.025*	

### Atomic displacement parameters (Å^2^)

**Table:**

	*U*^11^	*U*^22^	*U*^33^	*U*^12^	*U*^13^	*U*^23^
O1	0.0249 (13)	0.0254 (11)	0.0297 (12)	0.0044 (12)	0.0068 (11)	0.0020 (10)
N1	0.0305 (19)	0.0385 (18)	0.0326 (17)	0.0104 (17)	0.0064 (16)	0.0122 (15)
C1	0.0240 (18)	0.0180 (15)	0.0185 (14)	−0.0039 (15)	−0.0014 (14)	−0.0037 (12)
C2	0.0215 (17)	0.0213 (15)	0.0244 (16)	−0.0025 (15)	0.0041 (14)	−0.0062 (13)
C3	0.0257 (18)	0.0216 (15)	0.0216 (15)	−0.0037 (17)	0.0047 (15)	−0.0005 (13)
C4	0.0236 (17)	0.0226 (15)	0.0237 (15)	−0.0024 (16)	−0.0032 (15)	−0.0039 (13)
C5	0.0183 (17)	0.0272 (16)	0.0242 (15)	0.0003 (15)	0.0017 (14)	−0.0039 (14)
C6	0.0196 (17)	0.0233 (15)	0.0229 (15)	−0.0050 (15)	0.0028 (14)	−0.0033 (13)
C7	0.0227 (17)	0.0184 (15)	0.0256 (16)	−0.0021 (15)	0.0001 (15)	−0.0051 (13)
C8	0.0242 (18)	0.0152 (14)	0.0269 (15)	−0.0016 (15)	0.0052 (15)	−0.0045 (13)
C9	0.0203 (16)	0.0113 (13)	0.0278 (16)	−0.0040 (13)	0.0037 (14)	−0.0071 (12)
C10	0.0207 (16)	0.0121 (14)	0.0234 (15)	−0.0012 (13)	−0.0001 (14)	−0.0065 (11)
C11	0.0153 (15)	0.0220 (15)	0.0240 (15)	−0.0012 (15)	0.0005 (14)	−0.0075 (13)
C12	0.0202 (17)	0.0171 (14)	0.0225 (14)	−0.0037 (15)	−0.0026 (14)	−0.0074 (12)
C13	0.0234 (17)	0.0188 (15)	0.0281 (16)	−0.0019 (15)	−0.0033 (15)	−0.0065 (13)
C14	0.035 (2)	0.0181 (15)	0.0297 (17)	0.0039 (16)	−0.0075 (17)	−0.0003 (14)
C15	0.035 (2)	0.0226 (16)	0.0255 (16)	−0.0092 (17)	0.0008 (17)	0.0016 (13)
C16	0.0245 (18)	0.0296 (17)	0.0249 (16)	−0.0078 (17)	0.0015 (15)	−0.0075 (14)
C17	0.0198 (16)	0.0196 (14)	0.0196 (14)	−0.0024 (14)	0.0009 (14)	−0.0085 (12)
C18	0.0188 (16)	0.0220 (15)	0.0251 (15)	0.0022 (15)	0.0027 (14)	−0.0069 (13)
C19	0.0221 (17)	0.0152 (14)	0.0261 (15)	0.0084 (15)	−0.0014 (14)	−0.0020 (13)

### Geometric parameters (Å, °)

**Table:**

O1—C7	1.238 (4)	C9—H9A	0.9300
N1—C4	1.358 (4)	C10—C11	1.388 (4)
N1—H2N1	0.86 (4)	C10—C19	1.426 (5)
N1—H1N1	0.90 (4)	C11—C12	1.413 (4)
C1—C2	1.402 (4)	C11—H11A	0.9300
C1—C6	1.411 (5)	C12—C13	1.421 (4)
C1—C7	1.480 (4)	C12—C17	1.430 (5)
C2—C3	1.374 (4)	C13—C14	1.363 (5)
C2—H2A	0.9300	C13—H13A	0.9300
C3—C4	1.412 (5)	C14—C15	1.413 (5)
C3—H3A	0.9300	C14—H14A	0.9300
C4—C5	1.403 (4)	C15—C16	1.364 (5)
C5—C6	1.374 (4)	C15—H15A	0.9300
C5—H5A	0.9300	C16—C17	1.415 (4)
C6—H6A	0.9300	C16—H16A	0.9300
C7—C8	1.480 (4)	C17—C18	1.417 (4)
C8—C9	1.330 (4)	C18—C19	1.356 (4)
C8—H8A	0.9300	C18—H18A	0.9300
C9—C10	1.461 (4)	C19—H19A	0.9300
			
C4—N1—H2N1	120 (3)	C11—C10—C19	118.1 (3)
C4—N1—H1N1	123 (2)	C11—C10—C9	119.7 (3)
H2N1—N1—H1N1	117 (4)	C19—C10—C9	122.1 (3)
C2—C1—C6	117.4 (3)	C10—C11—C12	121.9 (3)
C2—C1—C7	119.2 (3)	C10—C11—H11A	119.0
C6—C1—C7	123.4 (3)	C12—C11—H11A	119.0
C3—C2—C1	121.7 (3)	C11—C12—C13	122.6 (3)
C3—C2—H2A	119.1	C11—C12—C17	118.9 (3)
C1—C2—H2A	119.1	C13—C12—C17	118.5 (3)
C2—C3—C4	120.6 (3)	C14—C13—C12	120.9 (3)
C2—C3—H3A	119.7	C14—C13—H13A	119.6
C4—C3—H3A	119.7	C12—C13—H13A	119.6
N1—C4—C5	121.5 (3)	C13—C14—C15	120.4 (3)
N1—C4—C3	120.6 (3)	C13—C14—H14A	119.8
C5—C4—C3	117.9 (3)	C15—C14—H14A	119.8
C6—C5—C4	121.1 (3)	C16—C15—C14	120.4 (3)
C6—C5—H5A	119.4	C16—C15—H15A	119.8
C4—C5—H5A	119.4	C14—C15—H15A	119.8
C5—C6—C1	121.2 (3)	C15—C16—C17	120.8 (3)
C5—C6—H6A	119.4	C15—C16—H16A	119.6
C1—C6—H6A	119.4	C17—C16—H16A	119.6
O1—C7—C8	119.6 (3)	C16—C17—C18	122.8 (3)
O1—C7—C1	121.0 (3)	C16—C17—C12	118.9 (3)
C8—C7—C1	119.4 (3)	C18—C17—C12	118.2 (3)
C9—C8—C7	121.6 (3)	C19—C18—C17	121.7 (3)
C9—C8—H8A	119.2	C19—C18—H18A	119.2
C7—C8—H8A	119.2	C17—C18—H18A	119.2
C8—C9—C10	126.7 (3)	C18—C19—C10	121.2 (3)
C8—C9—H9A	116.7	C18—C19—H19A	119.4
C10—C9—H9A	116.7	C10—C19—H19A	119.4
			
C6—C1—C2—C3	0.5 (4)	C9—C10—C11—C12	−176.3 (3)
C7—C1—C2—C3	−178.1 (3)	C10—C11—C12—C13	178.3 (3)
C1—C2—C3—C4	−0.7 (5)	C10—C11—C12—C17	−0.7 (4)
C2—C3—C4—N1	−179.2 (3)	C11—C12—C13—C14	−179.0 (3)
C2—C3—C4—C5	1.0 (5)	C17—C12—C13—C14	0.0 (4)
N1—C4—C5—C6	179.1 (3)	C12—C13—C14—C15	0.2 (5)
C3—C4—C5—C6	−1.0 (5)	C13—C14—C15—C16	−0.8 (5)
C4—C5—C6—C1	0.9 (5)	C14—C15—C16—C17	1.2 (5)
C2—C1—C6—C5	−0.6 (4)	C15—C16—C17—C18	178.7 (3)
C7—C1—C6—C5	178.0 (3)	C15—C16—C17—C12	−1.0 (5)
C2—C1—C7—O1	5.3 (4)	C11—C12—C17—C16	179.4 (3)
C6—C1—C7—O1	−173.3 (3)	C13—C12—C17—C16	0.4 (4)
C2—C1—C7—C8	−175.6 (3)	C11—C12—C17—C18	−0.3 (4)
C6—C1—C7—C8	5.8 (4)	C13—C12—C17—C18	−179.3 (3)
O1—C7—C8—C9	6.4 (5)	C16—C17—C18—C19	−178.9 (3)
C1—C7—C8—C9	−172.7 (3)	C12—C17—C18—C19	0.8 (4)
C7—C8—C9—C10	179.1 (3)	C17—C18—C19—C10	−0.3 (5)
C8—C9—C10—C11	165.8 (3)	C11—C10—C19—C18	−0.6 (4)
C8—C9—C10—C19	−11.5 (5)	C9—C10—C19—C18	176.8 (3)
C19—C10—C11—C12	1.1 (4)		

### Hydrogen-bond geometry (Å, °)

**Table:**

*Cg*1, *Cg*2 and *Cg*3 are the centroids of the C1–C6, C10–C12/C17–C19 and C12–C17 rings, respectively.

**Table:**

*D*—H···*A*	*D*—H	H···*A*	*D*···*A*	*D*—H···*A*
N1—H1N1···O1^i^	0.90 (4)	2.12 (4)	2.974 (4)	159 (4)
N1—H2N1···Cg1^ii^	0.86 (4)	2.99 (4)	3.475 (3)	118 (3)
C5—H5A···Cg3^iii^	0.93	2.82	3.513 (3)	132
C11—H11A···Cg3^iv^	0.93	2.92	3.631 (3)	135
C13—H13A···Cg2^iv^	0.93	2.86	3.551 (3)	132
C16—H16A···Cg1^iii^	0.93	2.87	3.603 (4)	136

Symmetry codes: (i) −*x*+2, *y*+1/2, −*z*+3/2; (ii) *x*+3/2, −*y*+1/2, −*z*+1; (iii) −*x*−1, *y*+3/2, −*z*+5/2; (iv) −*x*, *y*+1/2, −*z*+5/2.
